# Anorexia in Medicare Fee-for-Service Beneficiaries: A Claims-Based Analysis of Epidemiology and Mortality

**DOI:** 10.1007/s12603-023-1882-4

**Published:** 2023-01-16

**Authors:** Simon Dagenais, R. A. Fielding, S. Clark, C. Cantu, S. Prasad, J. D. Groarke

**Affiliations:** 1 Pfizer, New York, NY USA; 2 Nutrition, Exercise Physiology, and Sarcopenia Laboratory, Jean Mayer USDA Human Nutrition Research Center on Aging at Tufts University, Boston, MA USA; 3 Clarify Health, San Francisco, CA USA; 4 94 Hillcrest Parkway, Winchester, MA 01890 USA

**Keywords:** Loss of appetite, anorexia, older adults, Medicare claims, epidemiology

## Abstract

**Objectives:**

Loss of appetite in older adults can lead to malnutrition, weight loss, frailty, and death, but little is known about its epidemiology in the United States (US). The objective of this study was to estimate the annual prevalence and incidence of anorexia in older adults with Medicare fee-for-service (FFS) health insurance.

**Design:**

Retrospective and observational analysis of administrative health insurance claims data.

**Setting:**

This study included Medicare FFS claims from all settings (eg, hospital inpatient/outpatient, office, assisted living facility, skilled nursing facility, hospice, rehabilitation facility, home).

**Participants:**

This study included all individuals aged 65 to 115 years old with continuous Medicare FFS medical coverage (Parts A and/or B) for at least one 12-month period from October 1, 2015, to September 30, 2021 (ie, approximately 30 million individuals each year).

**Intervention:**

Not applicable.

**Measurements:**

Anorexia was identified using medical claims with the ICD-10 diagnosis code “R63.0: Anorexia”. This study compared individuals with anorexia to a control group without anorexia with respect to demographics, comorbidities using the Charlson Comorbidity Index (CCI), Claims-based Frailty Index (CFI), and annual mortality. The annual prevalence and incidence of anorexia were estimated for each 12-month period from October 1, 2015, to September 30, 2021.

**Results:**

The number of individuals with anorexia ranged from 317,964 to 328,977 per year, a mean annual prevalence rate of 1.1%. The number of individuals newly diagnosed with anorexia ranged from 243,391 to 281,071 per year, a mean annual incidence rate of 0.9%. Individuals with anorexia had a mean (±standard deviation) age of 80.5±8.7 years (vs 74.9±7.5 years without anorexia; p<.001), 64.4% were female (vs 53.8%; p<.001), and 78.4% were White (vs 83.2%; p<.001). The most common CCI comorbidities for those with anorexia were chronic pulmonary disease (39.4%), dementia (38.3%), and peripheral vascular disease (38.0%). Median (interquartile range [IQR]) CCI with anorexia was 4 [5] (vs 1 [3] without anorexia; p<.001). The annual mortality rate among those with anorexia was 22.3% (vs 4.1% without anorexia; relative risk 5.49 [95% confidence interval, 5.45–5.53]).

**Conclusion:**

Approximately 1% of all adults aged 65–115 years old with Medicare FFS insurance are diagnosed with anorexia each year based on ICD-10 codes reported in claims. These individuals have a higher comorbidity burden and an increased risk of annual mortality compared to those without a diagnosis of anorexia. Further analyses are needed to better understand the relationship between anorexia, comorbidities, frailty, mortality, and other health outcomes.

**Electronic Supplementary Material:**

Supplementary material is available for this article at 10.1007/s12603-023-1882-4 and is accessible for authorized users.

## Introduction

In older adults, loss of appetite (LOA) is multifactorial in etiology and may occur as a consequence of an underlying disease, as an adverse event of medications, or in the absence of a specific medical cause as part of the aging process (1–3). Factors that can contribute to the development of LOA include physiologic and metabolic changes with advancing age such as reductions in basal metabolic rate, shifts in body composition favoring increased fat mass and loss in skeletal muscle mass, changes to psychosocial functioning, multi-morbidity, and polypharmacy, as well as diminished senses of taste and smell (3, 4). If left untreated, LOA can lead to nutritional deficiencies, weight loss, frailty, sarcopenia, falls, osteoporosis, hip fracture, longer hospital stays, and increased morbidity and mortality (2, 5).

An estimated 21% to 30% of community-dwelling older adults suffer from LOA, with women more likely to experience LOA than men (6–9). In nursing homes, an estimated 31% of older adults have LOA, while in hospital settings, 32% to 42% of older adults have LOA (4, 10, 11). However, the methods for assessing appetite and identifying LOA vary between studies, making comparisons difficult and limiting our understanding of the true prevalence and incidence of LOA in older adults. In addition, few studies have used real-world data such as third-party health insurance claims (“claims”) to explore the burden of illness associated with LOA at a population level, and no study to our knowledge has explored the association between LOA and mortality in a large population of older adults in the US.

The objectives of the current analysis were to 1) describe the epidemiology (prevalence, incidence, trends) of LOA based on diagnosis codes reported in medical claims among adults 65 to 115 years old covered by the Medicare fee-for-service (FFS) health plan in the US, 2) examine comorbidities observed among adults aged 65 to 115 years old with a claim for LOA, and 3) explore mortality for adults 65–115 years old with a claim for LOA.

## Methods

### Study design

This was an observational, retrospective analysis using administrative claims data to characterize the annual prevalence and incidence of LOA diagnosed in FFS beneficiaries in the US aged 65 to 115 years old. The overall study period was from October 1, 2015 — when International Classification of Disease (ICD), 10th edition (ICD-10), diagnosis codes were mandated by the Centers for Medicare and Medicaid (CMS) in the US — to September 30, 2021. The overall study period was subdivided into six periods of 1 year each, with the first year (October 1, 2015 to September 30, 2016) labeled as “2016”, followed by subsequent years through 2021. Trends in annual prevalence and incidence were examined across these years. Comparisons with respect to demographics, comorbidities, frailty, and mortality between those with a diagnosis code for LOA and those without LOA were made using data from 2019, since this is the most recent year available prior to the coronavirus disease 2019 pandemic.

### Data source

Medical claims data for Medicare FFS beneficiaries were analyzed by Clarify Health (San Francisco, CA) in the Virtual Research Data Center from CMS. The Medicare FFS database contains medical, facility, professional, and prescription claims (Part A, institutional; Part B, non-institutional; Part D, drug event) for 100% of Medicare FFS beneficiaries. Claims include ICD-10 diagnosis and procedure codes, Current Procedural Terminology (CPT) procedure codes, Healthcare Common Procedure Coding System (HCPCS) procedure codes, dates of services, and additional information at the beneficiary level.

### Study populations

Eligibility criteria for the study population were 1) continuous coverage by FFS (Parts A and/or B) for medical benefits for at least 2 consecutive years and 2) 65 to 115 years old at the beginning of a given year; the upper age limit is suggested by CMS to minimize the impact of missing mortality data on calculated age (12). Individuals were identified as having LOA if they had at least 1 medical claim in any setting (eg, office, hospital outpatient) with the ICD-10 diagnosis code “R63.0: Anorexia” in any position (eg, primary, secondary). This diagnosis code describes a clinical disorder characterized by anorexia, lack of appetite, LOA, or dysorexia, but excludes LOA from eating disorders such as anorexia nervosa (ICD-10 codes F50.00, F50.01, F50.02) and avoidant or restrictive food intake disorder (ICD-10 code F50.81), as well as LOA of nonorganic origin (ICD-10 code F50.89) (13). The term “anorexia” is used to describe this disorder throughout the article. A control population of individuals without any claims for anorexia at any time during the study period was established as a comparator.

### Study measures

The primary outcomes for this analysis were the annual prevalence and incidence of anorexia as identified by the diagnosis code R63.0. In each year of the overall study period, the annual prevalence was calculated by dividing the number of individuals 65 to 115 years old with a claim for R63.0 in that year by the total number of individuals 65 to 115 years old who were continuously enrolled in Medicare FFS in that year. The annual incidence was calculated in the same way, except the numerator was the number of individuals 65 to 115 years old with a claim for R63.0 in that year who did not have a claim with the diagnosis code R63.0 in the prior year. Incidence was not calculated for 2016 because 12 months of observation was not available to establish new cases with anorexia. Annual prevalence and incidence were provided overall, by sex, and by 5-year age groups.

Individuals with a diagnosis code for anorexia were compared to a control population without anorexia in terms of comorbidities using the Charlson Comorbidity Index (CCI), a modified version of the Claims-based Frailty Index (CFI), and annual mortality. Patient age was assessed continuously in years and within 5-year age categories (65–69, 70–74, 75–79, 80–84, 85–89, 90–94, and ≥95). Other demographic characteristics included sex (male/female/unknown), race/ethnicity (White, Black, Hispanic, Asian, Other, Unknown/Missing), and US Census Region (Northeast, South, West, Midwest, Unknown/Missing). Demographic information was determined at the beginning of the given year for both populations.

### Comorbidities/frailty

Comorbidities were assessed using a modified CCI that subdivided renal disease into «mild/moderate» or «severe» (14, 15). Frailty was assessed using a modified CFI (16). The CFI was modified to remove “anorexia” as one of its components since all patients with a diagnosis code for anorexia met this requirement versus none in the control group.

### Mortality

Annual mortality was compared between individuals with a diagnosis code for anorexia versus the control population without anorexia. Mortality was based on the number of deaths during a given year among patients who were in each group in the previous year (eg, deaths in 2019 were for individuals with or without a diagnosis code for anorexia in 2018). Mortality was presented overall, by sex, and by 5-year age group.

### Statistical analysis

Categorical data were presented as frequencies with proportions, while continuous data were presented as means with standard deviations and medians with interquartile ranges, as appropriate. Per CMS restrictions, analyses containing subgroups with fewer than 11 individuals were suppressed. Differences between groups for categorical data were compared using Chi-square tests, while differences between groups for continuous data were analyzed using t-tests. Relative risks (RRs) with 95% confidence intervals were calculated to compare annual mortality rates for individuals with anorexia versus the control group. All statistical analyses were conducted using Statistical Analysis System (SAS) version 8.2 (SAS Institute, Cary, North Carolina); p<.05 was deemed statistically significant. The underlying SAS code and resulting data were reviewed by a second analyst at Clarify Health for quality control purposes.

As this analysis only used commercially available de-identified data, formal review and approval by an institutional review board/independent ethics committee was not sought.

## Results

### Annual prevalence

**Figure 1:**
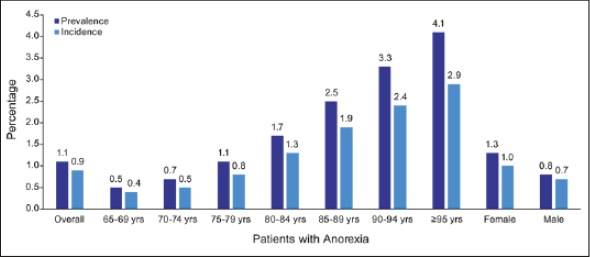
Prevalence and incidence of an ICD-10 diagnosis code for anorexia, overall and by age and sex, in the Medicare fee-for-service population in 2019 The anorexia population is all Medicare fee-for-service patients 65–115 years of age with continuous medical coverage that year (2019), no Part C enrollment, and an R63.0 diagnosis that year. Abbreviations: yrs, years of age

The total number of individuals with a diagnosis code for anorexia in a given year was consistent over the study period, ranging from 317,964 in 2016 to 326349 in 2021, resulting in a mean annual prevalence of 1.1% (Figure SI; Table SI). Across all years, anorexia increased in prevalence with age and was higher in females versus males (Figure 1; Table SI).

### Annual incidence

The total number of individuals newly diagnosed with anorexia in a given year—and no diagnosis of anorexia in the preceding year—was also consistent over the study period, ranging from 281,071 in 2017 to 247,085 in 2021, with a mean annual incidence rate of 0.9% (Figure SI; Table S2). In every year, the annual incidence rate was higher in females than in males and increased progressively in each 5-year age group, from 65–69 years to ≥95 years old (Figure 1; Table S2). Additional subgroup analyses are provided in Tables S3 and S4.

### Demographics

In 2019, there were 29,802,591 beneficiaries with Medicare FFS who met the study eligibility criteria, including 324213 individuals (1.1%) with a diagnosis code for anorexia and 29,478,378 (98.9%) individuals in the control population without anorexia (Table 1). Individuals with a diagnosis code for anorexia, compared to controls, were older, more likely to be female, less likely to be White, and more likely to be from the South (Table 1). The age distribution trended older in individuals with a diagnosis code for anorexia compared to the control population, overall and by female and male subgroups (Table 1;TableS5).

**Table 1:** Demographic characteristics of individuals with an ICD-10 diagnosis code for anorexia (N=29,802,591) in 2019

	**Anorexia population**^**a**^		**Control population**^**b**^		**p**
	**n**	%	**n**	%	
Total	324,213		29,478,378		
Age (mean±SD)	80.5±8.7		74.9±7.5		<.001
Age groups, years (%)					<.001
65–69	39,804	12.3	8,424,221	28.6	
70–74	54,594	16.8	8,355,619	28.3	
75–79	58,314	18.0	5,438,230	18.5	
80–84	60,232	18.6	3,537,787	12.0	
85–89	55,189	17.0	2,174,612	7.4	
90–94	37,989	11.7	1,119,142	3.8	
≥95	18,091	5.6	428,767	1.5	
Sex (%)					<.001
Female	208,772	64.4	15,859,790	53.8	
Male	115,441	35.6	13,618,585	46.2	
Unknown/Missing	0	0.0	<11	0.0	
Race/ethnicity (%)					<.001
Black	39,357	12.1	2,202,876	7.5	
White	254,114	78.4	24,537,178	83.2	
Hispanic	8,080	2.5	571,371	1.9	
Asian	11,522	3.6	720,923	2.5	
Other	7,541	2.3	742,771	2.5	
Unknown/Missing	3,599	1.1	703,259	2.4	
Region (%)					<.001
Midwest	67,100	20.7	6,295,646	21.4	
Northeast	51,236	15.8	5,275,720	17.9	
South	150,424	46.4	11,207,675	38.0	
West	54,689	16.9	6,112,909	20.7	
Unknown/Missing	764	0.2	586,428	2.0	

a. The anorexia population is all Medicare fee-for-services patients 65–115 years of age with continuous medical coverage that year (2019), no Part C enrollment, and an R63.0 diagnosis that year. b. The control population had no R63.0 diagnosis that year (2019) and no R63.0 diagnosis previously. Abbreviation: SD, standard deviation.

### Comorbidities

The median CCI score (IQR) was significantly higher for those with a diagnosis code for anorexia compared to the control population (4 [5] vs 1 [3]; p<.001; Table 2). Virtually all (93.9%) individuals with a diagnosis code for anorexia had also been diagnosed with 1 or more of the 17 CCI comorbidities during a 12-month period, compared with 58.1% in the control population (p<.001). Among those with a diagnosis code for anorexia, the most common CCI categories of comorbidities were chronic pulmonary disease (39.4% vs 17.8% for control), dementia (38.3% vs 6.6%), and peripheral vascular disease (38.0% vs 13.8%). Only 6.1% of individuals with a diagnosis code for anorexia had a CCI score of 0 (ie, no comorbidities), compared with 41.9% of controls; almost half (48.1%) of those with a diagnosis code for anorexia had a CCI score of ≥5, suggesting a severe burden of illness related to comorbidities, compared with only 14.4% of controls. When examining age distribution within each CCI severity score, the age distribution trended higher in each CCI severity category in those with a diagnosis code for anorexia compared to the control population (Table S6).

**Table 2:** Comparison of modified Charlson Comorbidity Index among individuals with or without an ICD-10 diagnosis code for anorexia in 2019

**Comorbidities**	**Anorexia population**^**a**^ **(n=324,213)**		**Control population**^**b**^ **(n=29,478,378)**		**p**
	**n**	**%**	**n**	**%**	**<.001**
AIDS/HIV	1,053	0.3	37,033	0.1	
Any malignancy, including leukemia and lymphoma	101,411	31.3	4,801,733	16.3	
Metastatic solid tumor	33,954	10.5	446,977	1.5	
Cerebrovascular disease	92,935	28.7	3,340,563	11.3	
Chronic pulmonary disease	127,676	39.4	5,233,471	17.8	
Dementia	124,213	38.3	1,948,465	6.6	
Diabetes with chronic complications	74,832	23.1	3,454,886	11.7	
Diabetes without chronic complications	118,015	36.4	6,970,127	23.6	
Congestive heart failure	104,463	32.2	3,168,946	10.8	
Mild liver disease	34,331	10.6	1,090,578	3.7	
Moderate or severe liver disease	3,720	1.1	88,265	0.3	
Myocardial infarction	45,425	14.0	1,387,583	4.7	
Peptic ulcer disease	19,470	6.0	364,814	1.2	
Peripheral vascular disease	123,250	38.0	4,068,817	13.8	
Mild or moderate renal disease	111,745	34.5	3,979,623	13.5	
Severe renal disease	23,484	7.2	479,273	1.6	
Rheumatologic disease	25,157	7.8	1,071,354	3.6	
Any of the above	304,314	93.9	17,115,790	58.1	<.001
CCI score					<.001
0	19,899	6.1	12,362,588	41.9	
1	32,974	10.2	4,516,248	15.3	
2	40,025	12.3	3,866,311	13.1	
3	39,996	12.3	2,784,574	9.4	
4	35,604	11.0	1,744,170	5.9	
5	30,424	9.4	1,320,760	4.5	
6	24,864	7.7	874,555	3.0	
7	19,839	6.1	612,306	2.1	
8	18,691	5.8	488,334	1.7	
9	16,676	5.1	339,998	1.2	
10	13,558	4.2	219,186	0.7	
11–19	31,326	9.7	347,415	1.2	
≥20	337	0.1	1,933	0.0	
Median (IQR)	4 (5)		1 (3)		<.001

a. The anorexia population is all Medicare fee-for-service patients 65–115 years of age with continuous medical coverage that year (2019), no Part C enrollment, and an R63.0 diagnosis that year. b. The control population had no R63.0 diagnosis that year (2019) and no R63.0 diagnosis previously. Abbreviations: AIDS/HIV, acquired immunodeficiency syndrome/human immunodeficiency virus; CCI, Charlson Comorbidity Index; IQR, interquartile range

### Frailty

A large majority (86.4%) of individuals with a diagnosis code for anorexia had a claim associated with 1 or more of the 17 comorbidities in the modified CFI, compared with only 26.7% in the control group (p<001; Table 3). Among those with a diagnosis code for anorexia, the most common CFI components were malaise and fatigue (51.6% vs 9.9% for control), transportation services (46.7% vs 10.2%). and muscle weakness (39.4% vs 8.0%). The proportion of individuals with ≥5 CFI components was much higher (29.8%) among those with a diagnosis code for anorexia than in the control population (3.0%; p<.001). Findings for both the CCI and modified CFI were consistent across each year from 2016 to 2021, with minimal variations over time (data not shown).

**Table 3:** Comparison of modified Claims-based Frailty Index among individuals with or without an ICD-10 diagnosis code for anorexia in 2019

**Frailty Index**	**Anorexia population**^**a**^ **(n=324,213)**		**Control population**^**b**^ **(n=29,478,378)**		**p**
	**n**	**%**	**n**	**%**	**<.001**
Abnormal loss of weight or underweight	125,635	38.8	831,376	2.8	
Abnormality of gait	95,613	29.5	2,136,933	7.2	
Accessories for oxygen delivery devices	23,097	7.1	688,308	2.3	
Adult failure to thrive	36,353	11.2	143,859	0.5	
Cachexia	15,706	4.8	65,612	0.2	
Debility	22,962	7.1	225,766	0.8	
Difficulty in walking	77,448	23.9	1,677,725	5.7	
History of fall	83,065	25.6	1,621,264	5.5	
Hospital beds and associated supplies	11,593	3.6	134,770	0.5	
Malaise and fatigue	167,374	51.6	2,905,722	9.9	
Muscle weakness	127,581	39.4	2,372,390	8.0	
Muscular wasting and disuse atrophy	9,887	3.0	102,383	0.3	
Pressure ulcer	38,444	11.9	454,166	1.5	
Senility without mention of psychosis	4,966	1.5	73,865	0.3	
Transportation services including ambulance	151,286	46.7	2,992,078	10.2	
Walking aids and attachments	23,806	7.3	600,560	2.0	
Wheelchairs, components, and accessories	23,942	7.4	372,378	1.3	
Any of the above	280,263	86.4	7,877,968	26.7	<.001
Number of Frailty Index conditions					<.001
0	43,950	13.6	21,600,410	73.3	
1	61,368	18.9	3,893,324	13.2	
2	47,742	14.7	1,642,559	5.6	
3	39,701	12.2	889,678	3.0	
4	35,063	10.8	574,089	1.9	
5	31,275	9.6	393,467	1.3	
6	25,548	7.9	251,472	0.9	
7	18,662	5.8	136,757	0.5	
8	11,253	3.5	62,121	0.2	
9	5,909	1.8	23,703	0.1	
≥10	3,742	1.2	10,798	0.0	
Median (IQR)	3 (4)		0 (1)		

a. The anorexia population is all Medicare fee-for-service patients 65–115 years of age with continuous medical coverage that year (2019), no Part C enrollment, and an R63.0 diagnosis that year. b. The control population had no R63.0 diagnosis that year (2019) and no R63.0 diagnosis previously. Abbreviation: IQR = interquartile range

### Mortality

**Figure 2:**
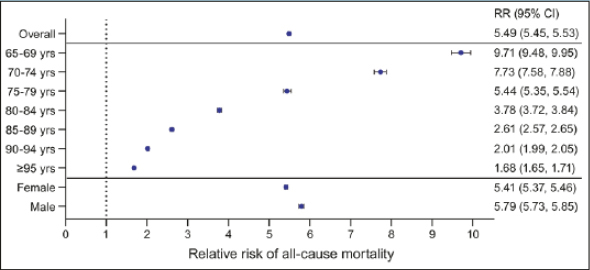
Relative risk (95% confidence interval) of mortality in individuals with an ICD-10 diagnosis code for anorexia in 2019 Deaths in 2019 are among those who were in the R63.0 or control populations in 2018. Abbreviations: CI, confidence interval; RR, relative risk; yrs, years of age

In 2019, overall annual mortality was significantly higher among individuals with a diagnosis code for anorexia (22.3%) than among controls (4.1%), with a RR of 5.49 (95% confidence interval, 5.45–5.53) (Figure 2; Table S7). Annual mortality was higher among individuals with a diagnosis code for anorexia compared to the control population across all age groups and for both females and males.

When examining subgroups, the annual mortality rate was consistently higher among females with anorexia versus female controls for all 5-year age groups, though the RR decreased from 10.92 for 65–69 years old to 1.68 for ≥95 years old (Table S8). A similar pattern was observed among subgroups of males by 5-year age groups, with a RR that decreased from 9.4 for 65–69 years old to 1.8 for ≥95 years old. These findings were generally consistent across each of the years from 2016 to 2021 (data not shown).

## Discussion

In this observational, retrospective analysis of administrative claims data in adults 65 to 115 years old with Medicare FFS coverage, a diagnosis code for anorexia was observed at least once in over 300,000 individuals during each year from 2016 to 2021, suggesting an annual prevalence of 1.1% for a diagnosis of anorexia among older adults in the US. This estimate is less than what would be expected based on studies reporting that anorexia is present in 21% to 42% of older adults (4, 6–11), suggesting that anorexia may currently be underdiagnosed by health care providers (HCPs) based on ICD-10 codes reported in medical claims in the US. Part of this discrepancy may be attributed to differences in study methods when defining and identifying individuals with anorexia, as previous studies relied on self-reported questionnaires or dietician review of food intake to identify anorexia. By contrast, the current study relied on claims data submitted by HCPs to CMS for the reimbursement of health care services for individuals with the ICD-10 diagnosis code R63.0. The lower estimates for the prevalence of anorexia identified in our analyses may be due to several important differences in case identification, including: 1) not all individuals identified as having anorexia according to self-reported questionnaires or dietician assessment will be diagnosed with anorexia by a HCP; 2) not all individuals diagnosed with anorexia by HCPs will have claims with the ICD-10 diagnosis code R63.0; and 3) not at all individuals diagnosed with anorexia will seek health care for anorexia.

These results are consistent with those of a recent cross-sectional study that examined claims-based surrogates of frailty in 3097 individuals ≥65 years old with Medicare coverage in the US; the study estimated the prevalence of anorexia—also based on the ICD-10 diagnosis code R63.0—at 0.5% in this population (16). When combined with our analyses, these findings suggest that 1% may be a conservative estimate of the lower bounds for the annual prevalence of anorexia among older adults with Medicare in the US.

The estimated annual incidence of 0.9% in our study suggests that a substantial number of individuals are newly diagnosed with anorexia by HCPs each year in the US, highlighting the burden of illness associated with this condition. Since the number of individuals with a diagnosis code for anorexia was consistent from 2016 to 2021, one hypothesis is that incident cases of anorexia may be replacing prevalent cases of anorexia who die each year, keeping the total number of individuals diagnosed with anorexia constant over time.

The prevalence and incidence of anorexia were higher among females compared to males in every age group over the age of 65 years. The reasons underlying this sex disparity are uncertain. Sex-based differences in baseline body mass index, body composition, nutritional status, threshold to self-report anorexia, and threshold for HCP to diagnose anorexia may contribute to this disparity.

Our analyses found that individuals with a diagnosis code for anorexia had multiple CCI comorbidities, suggesting much poorer overall health than those without a diagnosis code for anorexia. Although this difference in CCI may reflect the older mean age of individuals with anorexia, similar findings were also observed within each of the 5-year age subgroups, suggesting that age alone cannot explain this finding. While our analysis is unable to determine if there is a causal link between any of the observed CCI comorbidities and anorexia, some associations appear plausible (eg, individuals with dementia may have a higher risk of anorexia).

The higher prevalence for all 17 components to the CFI among individuals with a diagnosis code for anorexia compared with those without anorexia suggests that individuals with anorexia experience multiple concurrent contributors to frailty. The associations between different frailty comorbidities in the CFI (eg, anorexia and “Abnormal loss of weight or underweight”), as well as the impact of CFI components on health outcomes such as mortality, are unclear and should be explored in future analyses.

Our analyses suggest that 22.3% of those diagnosed with anorexia in the US die over a 12-month period, compared with only 4.1% for those without anorexia. To the best of our knowledge, this is the first study to report an association between anorexia and increased mortality in a US population; previous studies that reported such an association were conducted outside the US (6, 11, 17–32). This more than 5-fold increase in the annual mortality rate for those with anorexia likely cannot be attributed entirely to differences in age between these 2 groups because mortality was consistently higher for those with anorexia within each of the 5-year age groups in our analyses. The higher mortality is certainly likely, at least in part, to be related to the higher comorbidity burden observed in those with versus without an anorexia diagnosis, as described above.

Importantly, these data also highlight that the association of anorexia and mortality may differ according to age, and that any mortality risk with anorexia may be more significant in younger age groups (eg, 65–69, 70–74) within older populations. The RR of mortality among individuals 65 to 69 years old is almost 10-fold higher in those with a diagnosis code for anorexia versus those without; this higher RR diminishes in a stepwise manner with each increase in age group. This finding suggests that anorexia may require particular attention from HCPs when reported in sexagenarians and septuagenarians. The attenuation of mortality risk associated with anorexia in older age groups may be in part related to greater competing risks for mortality with advancing age (33). In addition, another potential explanation for this attenuation is that anorexia in octogenarians and nonagenarians may be more likely to reflect an age-related physiological change rather than a pathological process compared to anorexia diagnosed in younger individuals.

### Limitations

This study has several limitations. As with all observational studies based on administrative claims data, there is the possibility of inaccurate or incomplete data. Claims data are generated primarily for administrative reasons (ie, to facilitate the reimbursement of health care services by a third-party payer) and are therefore not intended for use in scientific research. Our analyses interpreted the ICD-10 diagnosis code “R63.0: Anorexia”, when used among adults ≥65 years old, as indicating the presence of anorexia, but this assumption has not been validated against more detailed clinical data (eg, electronic health records). Similarly, the absence of a diagnosis code for anorexia in claims does not exclude the possibility that an individual suffers from anorexia. The ICD-10 diagnosis codes reflected on claims could be inadvertently inaccurate (ie, data entry error), could represent working diagnoses at the time of the health care service that are subsequently ruled out after obtaining additional information, or may be influenced by reimbursement or other nonmedical considerations. Our study used 12-month observation periods, and individuals with anorexia were not followed over time to examine disease progression or determine the sequential order of CCI comorbidities or CFI components. It is possible that longer periods of observation (eg, 24/36 months) are more appropriate to better understand the burden of illness associated with anorexia. This study was not designed to evaluate causal associations between anorexia and CCI, CFI, or mortality.

Nevertheless, this study does contribute to the scientific literature on the epidemiology of anorexia among older adults in the US, which has been very limited to date. Our study used a large, national sample that represents 100% of the Medicare FFS population in the US (and approximately 60% of all Medicare beneficiaries in the US) (34), with nearly 30 million participants included per year. Our study period was 72 months and included 6 separate 12-month intervals; this allowed us to compare findings across multiple time intervals to ensure internal consistency over time. Our findings of an increased burden of CCI comorbidities, CFI components, and increased annual mortality rate for individuals with anorexia are also consistent with findings from previous studies, which have shown that anorexia is associated with worse health outcomes, including an increased risk of sarcopenia, disability, hospital acquired infection, and mortality (6–8, 11, 30).

### Opportunities for future analyses

Our study raised several interesting findings that should be explored in future analyses, including differences observed for individuals with a diagnosis code for anorexia, versus those without, with respect to 1) mean age, 2) sex distribution, 3) race, 4) geographic region, and 5) annual mortality in younger age groups. Additional analyses are also needed to understand differences noted between females and males with anorexia, including a higher 1) annual prevalence of anorexia in females versus males, 2) annual incidence in females versus males, and 3) risk of annual mortality in males versus females. Additional analyses are also needed to establish the timing and relationship of diagnoses for anorexia, CCI comorbidities, CFI components, and mortality.

## Conclusions

The results of this large, observational study based on administrative claims data suggest that although anorexia is diagnosed in many Medicare FFS beneficiaries every year, anorexia is likely underdiagnosed based on ICD-10 codes reported in medical claims for older adults in the US. The burden of comorbidities, including those associated with frailty, is significantly higher among older adults with a diagnosis code for anorexia compared to those without. The RR of mortality in those ≥65 years old is significantly increased for those with a diagnosis code for anorexia, though this RR decreases progressively with advancing age. The high burden of illness for individuals with anorexia compared to those without anorexia underscores the importance of diagnosing and treating this condition. More studies are needed to further elucidate the causes, impact, and optimal treatment of anorexia.

## Electronic Supplementary Material

Supplementary material, approximately 112 KB.
