# Supplementary material for: Anorexia in Medicare Fee-for-Service Beneficiaries: A Claims-Based Analysis of Epidemiology and Mortality
Source: J Nutr Health Aging. 2023 Jan 16:1–8. Online ahead of print. doi: 10.1007/s12603-023-1882-4 (PMC9841141; doi:10.1007/s12603-023-1882-4)
Supplement: Supplementary file 1 — Supplementary material, approximately 112 KB. [file 12603_2023_1882_MOESM1_ESM.docx]

**SUPPLEMENT**

FIGURE S1. Prevalence and incidence rates of an ICD-10 diagnosis code for anorexia over time (95% confidence interval)

**
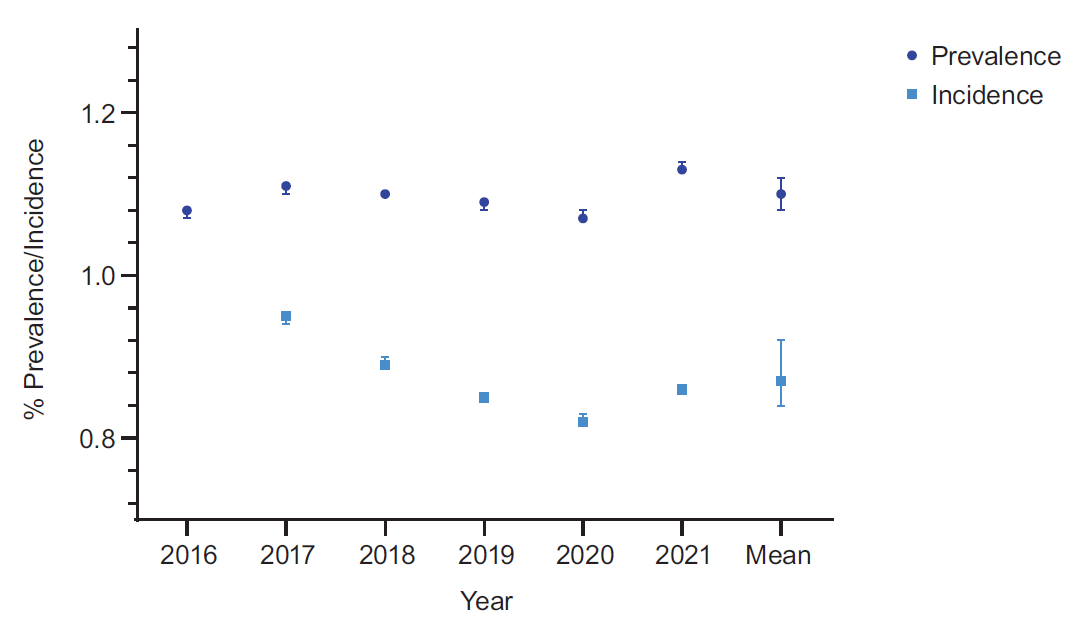
**

|  | | **2016** | | **2017** | | **2018** | | **2019** | | **2020** | | **2021** | | **Mean** | |
| --- | --- | --- | --- | --- | --- | --- | --- | --- | --- | --- | --- | --- | --- | --- | --- |
| **Group** |  | ***n*** | **%** | ***n*** | **%** | ***n*** | **%** | ***n*** | **%** | ***n*** | **%** | ***n*** | **%** | ***n*** | **%** |
| **65-69** | **Numerator** | 38,371 | 0.4 | 39,402 | 0.5 | 39,737 | 0.5 | 39,804 | 0.5 | 39,412 | 0.5 | 39,773 | 0.5 | 39,417 | 0.5 |
|  | **Denominator** | 8,624,465 |  | 8,516,822 |  | 8,510,070 |  | 8,464,025 |  | 8,370,217 |  | 7,926,198 |  | 8,401,966 |  |
| **70-74** | **Numerator** | 49,096 | 0.6 | 52,757 | 0.7 | 53,434 | 0.7 | 54,594 | 0.7 | 54,669 | 0.6 | 58,222 | 0.7 | 53,795 | 0.7 |
|  | **Denominator** | 7,642,863 |  | 8,027,024 |  | 8,206,032 |  | 8,410,213 |  | 8,554,578 |  | 8,305,383 |  | 8,191,016 |  |
| **75-79** | **Numerator** | 55,541 | 1.1 | 57,995 | 1.1 | 58,365 | 1.1 | 58,314 | 1.1 | 56,806 | 1.0 | 61,047 | 1.1 | 58,011 | 1.1 |
|  | **Denominator** | 5,254,890 |  | 5,380,586 |  | 5,463,002 |  | 5,496,544 |  | 5,478,708 |  | 5,544,455 |  | 5,436,364 |  |
| **80-84** | **Numerator** | 59,634 | 1.6 | 61,071 | 1.7 | 61,283 | 1.7 | 60,232 | 1.7 | 58,789 | 1.7 | 60,077 | 1.7 | 60,181 | 1.7 |
|  | **Denominator** | 3,697,725 |  | 3,644,776 |  | 3,636,032 |  | 3,598,019 |  | 3,564,680 |  | 3,477,215 |  | 3,603,075 |  |
| **85-89** | **Numerator** | 58,874 | 2.3 | 59,056 | 2.4 | 56,689 | 2.5 | 55,189 | 2.5 | 52,589 | 2.4 | 53,324 | 2.6 | 55,954 | 2.5 |
|  | **Denominator** | 2,533,597 |  | 2,419,559 |  | 2,302,102 |  | 2,229,801 |  | 2,159,879 |  | 2,056,188 |  | 2,283,521 |  |
| **90-94** | **Numerator** | 39,302 | 3.0 | 40,866 | 3.3 | 39,904 | 3.3 | 37,989 | 3.3 | 37,635 | 3.4 | 36,267 | 3.5 | 38,661 | 3.3 |
|  | **Denominator** | 1,294,147 |  | 1,251,140 |  | 1,204,865 |  | 1,157,131 |  | 1,120,160 |  | 1,045,835 |  | 1,178,880 |  |
| **≥95** | **Numerator** | 17,146 | 3.7 | 17,830 | 3.9 | 18,304 | 4.1 | 18,091 | 4.1 | 18,176 | 4.2 | 17,639 | 4.3 | 17,864 | 4.0 |
|  | **Denominator** | 469,660 |  | 461,810 |  | 451,830 |  | 446,858 |  | 436,390 |  | 415,061 |  | 446,935 |  |
| **Male** | **Numerator** | 109,419 | 0.8 | 114,851 | 0.9 | 115,427 | 0.9 | 115,441 | 0.8 | 112,908 | 0.8 | 116,910 | 0.9 | 114,159 | 0.8 |
|  | **Denominator** | 13,364,008 |  | 13,534,742 |  | 13,644,986 |  | 13,734,026 |  | 13,723,196 |  | 13,315,105 |  | 13,552,677 |  |
| **Female** | **Numerator** | 208,545 | 1.3 | 214,126 | 1.3 | 212,289 | 1.3 | 208,772 | 1.3 | 205,168 | 1.3 | 209,439 | 1.4 | 209,723 | 1.3 |
|  | **Denominator** | 16,153,337 |  | 16,166,972 |  | 16,128,943 |  | 16,068,562 |  | 15,961,412 |  | 15,455,226 |  | 15,989,075 |  |
| **All Patients** | **Numerator** | 317,964 | 1.1 | 328,977 | 1.1 | 327,716 | 1.1 | 324,213 | 1.1 | 318,076 | 1.1 | 326,349 | 1.1 | 323,883 | 1.1 |
|  | **Denominator** | 29,517,347 |  | 29,701,717 |  | 29,773,933 |  | 29,802,591 |  | 29,684,612 |  | 28,770,335 |  | 29,541,756 |  |

TABLE S1. Prevalence rates of an ICD-10 diagnosis code for anorexia over time

TABLE S2. Incidence rates of an ICD-10 diagnosis code for anorexia over time

|  | | **2017** | | **2018** | | **2019** | | **2020** | | **2021** | | **Mean** | |
| --- | --- | --- | --- | --- | --- | --- | --- | --- | --- | --- | --- | --- | --- |
| **Group** |  | ***n*** | **%** | ***n*** | **%** | ***n*** | **%** | ***n*** | **%** | ***n*** | **%** | ***n*** | **%** |
| **65-69** | **Numerator** | 35,068 | 0.4 | 34,054 | 0.4 | 33,366 | 0.4 | 32,945 | 0.4 | 33,105 | 0.4 | 33,708 | 0.4 |
|  | **Denominator** | 8,512,488 |  | 8,504,387 |  | 8,457,587 |  | 8,363,750 |  | 7,919,530 |  | 8,351,548 |  |
| **70-74** | **Numerator** | 46,382 | 0.6 | 45,010 | 0.6 | 44,648 | 0.5 | 43,822 | 0.5 | 46,666 | 0.6 | 45,306 | 0.6 |
|  | **Denominator** | 8,020,649 |  | 8,197,608 |  | 8,400,267 |  | 8,543,731 |  | 8,293,827 |  | 8,291,216 |  |
| **75-79** | **Numerator** | 50,322 | 0.9 | 48,186 | 0.9 | 46,533 | 0.8 | 44,372 | 0.8 | 47,479 | 0.9 | 47,378 | 0.9 |
|  | **Denominator** | 5,372,913 |  | 5,452,823 |  | 5,484,763 |  | 5,466,274 |  | 5,530,887 |  | 5,461,532 |  |
| **80-84** | **Numerator** | 52,002 | 1.4 | 49,428 | 1.4 | 46,590 | 1.3 | 44,539 | 1.3 | 44,920 | 1.3 | 47,496 | 1.3 |
|  | **Denominator** | 3,635,707 |  | 3,624,177 |  | 3,584,377 |  | 3,550,430 |  | 3,462,058 |  | 3,571,350 |  |
| **85-89** | **Numerator** | 49,461 | 2.1 | 44,554 | 2.0 | 41,427 | 1.9 | 38,461 | 1.8 | 38,380 | 1.9 | 42,457 | 1.9 |
|  | **Denominator** | 2,409,964 |  | 2,289,967 |  | 2,216,039 |  | 2,145,751 |  | 2,041,244 |  | 2,220,593 |  |
| **90-94** | **Numerator** | 33,686 | 2.7 | 30,642 | 2.6 | 27,654 | 2.4 | 26,802 | 2.4 | 25,012 | 2.4 | 28,759 | 2.5 |
|  | **Denominator** | 1,243,960 |  | 1,195,603 |  | 1,146,796 |  | 1,109,327 |  | 1,034,580 |  | 1,146,053 |  |
| **≥95** | **Numerator** | 14,150 | 3.1 | 13,589 | 3.0 | 12,591 | 2.9 | 12,450 | 2.9 | 11,523 | 2.8 | 12,861 | 2.9 |
|  | **Denominator** | 458,130 |  | 447,115 |  | 441,358 |  | 430,664 |  | 408,945 |  | 437,242 |  |
| **Male** | **Numerator** | 100,831 | 0.8 | 96,620 | 0.7 | 93,737 | 0.7 | 89,997 | 0.7 | 92,670 | 0.7 | 94,771 | 0.7 |
|  | **Denominator** | 13,520,722 |  | 13,626,179 |  | 13,712,322 |  | 13,700,285 |  | 13,290,865 |  | 13,570,075 |  |
| **Female** | **Numerator** | 180,240 | 1.1 | 168,843 | 1.1 | 159,072 | 1.0 | 153,394 | 1.0 | 154,415 | 1.0 | 163,193 | 1.0 |
|  | **Denominator** | 16,133,086 |  | 16,085,497 |  | 16,018,862 |  | 15,909,638 |  | 15,400,202 |  | 15,909,457 |  |
| **All Patients** | **Numerator** | 281,071 | 1.0 | 265,463 | 0.9 | 252,809 | 0.9 | 243,391 | 0.8 | 247,085 | 0.9 | 257,964 | 0.9 |
|  | **Denominator** | 29,653,811 |  | 29,711,680 |  | 29,731,187 |  | 29,609,927 |  | 28,691,071 |  | 29,479,535 |  |

TABLE S3. Prevalence rates of an ICD-10 diagnosis code for anorexia by subgroups of sex and age over time

|  |  | **2016** | **2017** | **2018** | **2019** | **2020** | **2021** | **Mean** |
| --- | --- | --- | --- | --- | --- | --- | --- | --- |
| **Sex** | **Age group, years** | **%** | **%** | **%** | **%** | **%** | **%** | **%** |
| **Female** | **65-69** | 0.5 | 0.5 | 0.5 | 0.5 | 0.5 | 0.6 | 0.5 |
|  | **70-74** | 0.7 | 0.8 | 0.7 | 0.7 | 0.7 | 0.8 | 0.8 |
|  | **75-79** | 1.2 | 1.2 | 1.2 | 1.2 | 1.2 | 1.3 | 1.2 |
|  | **80-84** | 1.8 | 1.9 | 1.9 | 1.9 | 1.9 | 2.0 | 1.9 |
|  | **85-89** | 2.6 | 2.8 | 2.8 | 2.8 | 2.8 | 3.0 | 2.8 |
|  | **90-94** | 3.3 | 3.6 | 3.7 | 3.6 | 3.8 | 3.9 | 3.6 |
|  | **≥95** | 3.9 | 4.2 | 4.4 | 4.4 | 4.6 | 4.7 | 4.4 |
| **Male** | **65-69** | 0.4 | 0.4 | 0.4 | 0.4 | 0.4 | 0.4 | 0.4 |
|  | **70-74** | 0.6 | 0.6 | 0.6 | 0.6 | 0.6 | 0.6 | 0.6 |
|  | **75-79** | 0.9 | 0.9 | 0.9 | 0.9 | 0.9 | 0.9 | 0.9 |
|  | **80-84** | 1.3 | 1.4 | 1.4 | 1.4 | 1.4 | 1.4 | 1.4 |
|  | **85-89** | 1.9 | 2.0 | 2.0 | 2.0 | 1.9 | 2.1 | 2.0 |
|  | **90-94** | 2.4 | 2.6 | 2.6 | 2.6 | 2.6 | 2.7 | 2.6 |
|  | **≥95** | 2.8 | 2.9 | 3.0 | 3.0 | 3.0 | 3.0 | 2.9 |

TABLE S4. Incidence rates of an ICD-10 diagnosis code for anorexia by subgroups of sex and age over time

|  |  | **2017** | **2018** | **2019** | **2020** | **2021** | **Mean** |
| --- | --- | --- | --- | --- | --- | --- | --- |
| **Sex** | **Age group, years** | **%** | **%** | **%** | **%** | **%** | **%** |
| **Female** | **65-69** | 0.5 | 0.4 | 0.4 | 0.4 | 0.5 | 0.5 |
|  | **70-74** | 0.7 | 0.6 | 0.6 | 0.6 | 0.6 | 0.6 |
|  | **75-79** | 1.1 | 1.0 | 1.0 | 0.9 | 1.0 | 1.0 |
|  | **80-84** | 1.6 | 1.5 | 1.4 | 1.4 | 1.5 | 1.5 |
|  | **85-89** | 2.3 | 2.2 | 2.1 | 2.0 | 2.1 | 2.1 |
|  | **90-94** | 2.9 | 2.8 | 2.6 | 2.7 | 2.6 | 2.7 |
|  | **≥95** | 3.3 | 3.3 | 3.1 | 3.1 | 3.1 | 3.2 |
| **Male** | **65-69** | 0.4 | 0.4 | 0.4 | 0.4 | 0.4 | 0.4 |
|  | **70-74** | 0.5 | 0.5 | 0.5 | 0.5 | 0.5 | 0.5 |
|  | **75-79** | 0.8 | 0.8 | 0.7 | 0.7 | 0.7 | 0.8 |
|  | **80-84** | 1.2 | 1.2 | 1.1 | 1.1 | 1.1 | 1.1 |
|  | **85-89** | 1.7 | 1.6 | 1.6 | 1.5 | 1.6 | 1.6 |
|  | **90-94** | 2.3 | 2.1 | 2.0 | 2.0 | 2.0 | 2.1 |
|  | **≥95** | 2.4 | 2.4 | 2.3 | 2.2 | 2.2 | 2.3 |

TABLE S5. Individuals with or without an ICD-10 diagnosis code for anorexia by sex and age in 2019

|  |  | **Anorexia**  **population^a^** | | **Control**  **population^b^** | |
| --- | --- | --- | --- | --- | --- |
| **Sex** | **Age group, years** | ***n*** | **%** | ***n*** | **%** |
| **Female** | **Total** | 208,772 | 100 | 15,859,790 | 100 |
|  | **65-69** | 22,322 | 10.7 | 4,293,652 | 27.1 |
|  | **70-74** | 32,084 | 15.4 | 4,287,838 | 27.0 |
|  | **75-79** | 35,554 | 17.0 | 2,916,392 | 18.4 |
|  | **80-84** | 38,552 | 18.5 | 1,996,127 | 12.6 |
|  | **85-89** | 37,774 | 18.1 | 1,309,943 | 8.3 |
|  | **90-94** | 27,989 | 13.4 | 742,835 | 4.7 |
|  | **≥95** | 14,497 | 6.9 | 313,003 | 2.0 |
| **Male** | **Total** | 115,441 | 100 | 13,618,585 | 100 |
|  | **65-69** | 17,482 | 15.1 | 4,130,568 | 30.3 |
|  | **70-74** | 22,510 | 19.5 | 4,067,781 | 29.9 |
|  | **75-79** | 22,760 | 19.7 | 2,521,837 | 18.5 |
|  | **80-84** | 21,680 | 18.8 | 1,541,660 | 11.3 |
|  | **85-89** | 17,415 | 15.1 | 864,668 | 6.3 |
|  | **90-94** | 10,000 | 8.7 | 376,307 | 2.8 |
|  | **≥95** | 3,594 | 3.1 | 115,764 | 0.9 |

^a^The anorexia population is all Medicare fee-for-service patients 65-115 years of age with continuous
medical coverage that year (2019), no Part C enrollment, and an R63.0 diagnosis that year.

^b^The control population had no R63.0 diagnosis that year (2019) and no R63.0 diagnosis previously.

TABLE S6. Comparison of individuals with or without an ICD-10 diagnosis code for anorexia by
modified Charlson Comorbidity Index severity score and age in 2019

|  |  | **Anorexia**  **population^a^** | | **Control**  **population^b^** | |
| --- | --- | --- | --- | --- | --- |
| **Modified CCI severity score** | **Age group, years** | ***n*** | **%** | ***n*** | **%** |
| **None** | **Total** | 19,902 | 100.0 | 12,362,588 | 100.0 |
|  | **65-69** | 4,256 | 21.4 | 4,764,020 | 38.5 |
|  | **70-74** | 4,670 | 23.5 | 3,870,595 | 31.3 |
|  | **75-79** | 3,632 | 18.2 | 1,905,323 | 15.4 |
|  | **80-84** | 3,138 | 15.8 | 981,679 | 7.9 |
|  | **85-89** | 2,203 | 11.1 | 506,933 | 4.1 |
|  | **90-94** | 1,401 | 7.0 | 228,842 | 1.9 |
|  | **≥95** | 599 | 3.0 | 105,196 | 0.9 |
| **Mild (1-2)** | **Total** | 72,999 | 100.0 | 8,382,559 | 100.0 |
|  | **65-69** | 9,735 | 13.3 | 2,130,749 | 25.4 |
|  | **70-74** | 12,872 | 17.6 | 2,404,968 | 28.7 |
|  | **75-79** | 12,832 | 17.6 | 1,670,501 | 19.9 |
|  | **80-84** | 12,724 | 17.4 | 1,078,327 | 12.9 |
|  | **85-89** | 11,753 | 16.1 | 644,465 | 7.7 |
|  | **90-94** | 8,562 | 11.7 | 331,425 | 4.0 |
|  | **≥95** | 4,521 | 6.2 | 122,124 | 1.5 |
| **Moderate (3-4)** | **Total** | 75,600 | 100.0 | 4,528,744 | 100.0 |
|  | **65-69** | 7,637 | 10.1 | 850,065 | 18.8 |
|  | **70-74** | 11,068 | 14.6 | 1,132,278 | 25.0 |
|  | **75-79** | 12,627 | 16.7 | 957,536 | 21.1 |
|  | **80-84** | 14,207 | 18.8 | 724,847 | 16.0 |
|  | **85-89** | 14,042 | 18.6 | 489,699 | 10.8 |
|  | **90-94** | 10,489 | 13.9 | 271,325 | 6.0 |
|  | **≥95** | 5,530 | 7.3 | 102,994 | 2.3 |
| **Severe (5+)** | **Total** | 155,715 | 100.0 | 4,204,487 | 100.0 |
|  | **65-69** | 18,176 | 11.7 | 679,387 | 16.2 |
|  | **70-74** | 25,984 | 16.7 | 947,778 | 22.5 |
|  | **75-79** | 29,223 | 18.8 | 904,870 | 21.5 |
|  | **80-84** | 30,163 | 19.4 | 752,934 | 17.9 |
|  | **85-89** | 27,191 | 17.5 | 533,515 | 12.7 |
|  | **90-94** | 17,537 | 11.3 | 287,550 | 6.8 |
|  | **≥95** | 7,441 | 4.8 | 98,453 | 2.3 |

^a^The anorexia population is all Medicare fee-for-service patients 65-115 years of age with continuous
medical coverage that year (2019), no Part C enrollment, and an R63.0 diagnosis that year.

^b^The control population had no R63.0 diagnosis that year (2019) and no R63.0 diagnosis previously.

Abbreviation: CCI, Charlson Comorbidity Index

TABLE S7. Mortality rates of individuals with and without an ICD-10 diagnosis code for anorexia in 2019

|  | **Anorexia population^a^** | | | **Control population^b^** | | | **Relative risk (95% CI)** |
| --- | --- | --- | --- | --- | --- | --- | --- |
|  | **Deaths** | **At risk** | **Incidence (%)** | **Deaths** | **At risk** | **Incidence (%)** |  |
| **Total** | 72,933 | 327,716 | 22.3% | 1,193,495 | 29,446,217 | 4.1% | 5.49 (5.45, 5.53) |
|  |  |  |  |  |  |  |  |
| **Age group, years (%)** |  |  |  |  |  |  |  |
| 65-69 | 5,966 | 39,737 | 15.0% | 130,963 | 8,470,333 | 1.5% | 9.71 (9.48, 9.95) |
| 70-74 | 8,742 | 53,434 | 16.4% | 172,538 | 8,152,598 | 2.1% | 7.73 (7.58, 7.88) |
| 75-79 | 10,793 | 58,365 | 18.5% | 183,599 | 5,404,637 | 3.4% | 5.44 (5.35, 5.54) |
| 80-84 | 13,099 | 61,283 | 21.4% | 202,399 | 3,574,749 | 5.7% | 3.78 (3.72, 3.84) |
| 85-89 | 14,387 | 56,689 | 25.4% | 218,106 | 2,245,413 | 9.7% | 2.61 (2.57, 2.65) |
| 90-94 | 12,747 | 39,904 | 31.9% | 184,502 | 1,164,961 | 15.8% | 2.01 (1.99, 2.05) |
| ≥95 | 7,199 | 18,304 | 39.3% | 101,388 | 433,526 | 23.4% | 1.68 (1.65, 1.71) |
| **Sex (%)** |  |  |  |  |  |  |  |
| Female | 44,201 | 212,289 | 20.8% | 612,244 | 15,916,654 | 3.8% | 5.41 (5.37, 5.46) |
| Male | 28,732 | 115,427 | 24.9% | 581,251 | 13,529,559 | 4.3% | 5.79 (5.73, 5.85) |

Deaths in 2019 are among those who were in the R63.0 or control populations in the previous year (2018).

^a^The anorexia population is all Medicare fee-for-service patients 65-115 years of age with continuous
medical coverage that year (2019), no Part C enrollment, and an R63.0 diagnosis that year.

^b^The control population had no R63.0 diagnosis that year (2019) and no R63.0 diagnosis previously.

Abbreviation: CI, confidence interval

TABLE S8. Mortality rates of individuals with and without an ICD-10 diagnosis code for anorexia by sex and age in 2019

|  |  | **Anorexia population^a^** | | | **Control population^b^** | | | **Relative risk (95% CI)** |
| --- | --- | --- | --- | --- | --- | --- | --- | --- |
| **Sex** | **Age group, years** | **Deaths** | **At risk** | **Incidence (%)** | **Deaths** | **At risk** | **Incidence (%)** |  |
| **Female** | **65-69** | 2,927 | 22,602 | 13.0% | 51,163 | 4,313,801 | 1.2% | 10.92 (10.55-11.31) |
|  | **70-74** | 4,431 | 31,493 | 14.1% | 71,236 | 4,199,293 | 1.7% | 8.30 (8.07-8.54) |
|  | **75-79** | 5,783 | 35,529 | 16.3% | 82,685 | 2,914,354 | 2.8% | 5.74 (5.60-5.88) |
|  | **80-84** | 7,459 | 39,350 | 19.0% | 99,771 | 2,026,675 | 4.9% | 3.86 (3.77-3.94) |
|  | **85-89** | 9,058 | 39,078 | 23.2% | 119,170 | 1,364,198 | 8.7% | 2.66 (2.61-2.71) |
|  | **90-94** | 8,884 | 29,520 | 30.1% | 114,940 | 779,940 | 14.7% | 2.05 (2.01-2.08) |
|  | **≥95** | 5,659 | 14,717 | 38.5% | 73,279 | 318,393 | 23.0% | 1.68 (1.64-1.71) |
|  | **Total** | **44,201** | **212,289** | **20.8%** | **612,244** | **15,916,654** | **3.8%** | **5.41 (5.37, 5.46)** |
| **Male** | **65-69** | 3,039 | 17,135 | 17.7% | 79,800 | 4,156,531 | 1.9% | 9.24 (8.94-9.55) |
|  | **70-74** | 4,311 | 21,941 | 19.6% | 101,302 | 3,953,304 | 2.6% | 7.67 (7.47-7.89) |
|  | **75-79** | 5,010 | 22,836 | 21.9% | 100,914 | 2,490,282 | 4.1% | 5.42 (5.28-5.56) |
|  | **80-84** | 5,640 | 21,933 | 25.7% | 102,628 | 1,548,074 | 6.6% | 3.88 (3.79-3.98) |
|  | **85-89** | 5,329 | 17,611 | 30.3% | 98,936 | 881,214 | 11.2% | 2.70 (2.64-2.76) |
|  | **90-94** | 3,863 | 10,384 | 37.2% | 69,562 | 385,021 | 18.1% | 2.06 (2.01-2.12) |
|  | **≥95** | 1,540 | 3,587 | 42.9% | 28,109 | 115,133 | 24.4% | 1.76 (1.70-1.83) |
|  | **Total** | **28,732** | **115,427** | **24.9%** | **581,251** | **13,529,559** | **4.3%** | **5.79 (5.73, 5.85)** |

Deaths in 2019 are among those who were in the R63.0 or control populations in the previous year (2018).

^a^The anorexia population is all Medicare fee-for-service patients 65-115 years of age with continuous medical
coverage that year (2019), no Part C enrollment, and an R63.0 diagnosis that year.

^b^The control population had no R63.0 diagnosis that year (2019) and no R63.0 diagnosis previously. In 2018, <11 individuals
in the control population had a missing or unknown sex characteristic.

Abbreviation: CI, confidence interval
